# Supplementary material for: Rose Virome Analysis and Identification of a Novel Ilarvirus in Taiwan
Source: Viruses. 2022 Nov 16;14(11):2537. doi: 10.3390/v14112537 (PMC9693529; doi:10.3390/v14112537)
Supplement: Supplementary file 1 [file viruses-14-02537-s001.zip › Table S1_Primers for virus detection-R2.pdf]

Table S1. Primers designed for virus detection.

Virus	Primer name	Primer sequence (5' → 3')	Product size (bp)	Target gene
Apple mosaic virus (ApMV)	AMV-R3-137-F	TGTTAACCTCTTACGATTTCGAG	1025	Full-length MP
	AMV-R3-1161-R	CTCCAGGGTGAGTGTGACCAC		
	AMV-R3-940-F	CGAAGATGTCTCCGATGGAAAGC	872	Full-length CP
	AMV-R3-1811-R	CACGATATTCGATCCCTGACCT		
Prunus necrotic ringspot virus (PNRSV)	PNRSV-MPF1[a]	GTTGGTTGAATAGTGTTTCAGTATGGCC	983	Full-length MP
	PNRSV-MPR1[a]	AGCGTGGGTATGATTGCAAATTCGG		
	PNRSV-CPF1[b]	ATGGTTTGCCGAATTTGCAATCAT	699	Full-length CP
	PNRSV-CPR1[b]	GAGTGTGCTTATCTCACTCTAG		
Rose ilarvirus 2 (RIV-2)	RIV2-R3-1F	CGCTGAAGCTGTTATCCTACCTGA	1080	Full-length MP
	RIV2-R3-1R	TGGAGAAAGGGACGCAAGATAGAG		
	RIV2-R3-2F	GGAACTGTCAACCGCTTCGAGGA	871	Full-length CP
	RIV2-R3-2R	AGCGCAAAAGCAATGACAGTACGG		
Apple stem grooving virus (ASGV)	ASGV140-F	GTGACTAACCGCTTCTTCTCTT	695	ORF1
	ASGV834-R	TGTAGCTTGGTCACCCTAATCTT		
Rose spring dwarf-associated virus (RSDaV)	RSDaV-F	CAGGCTCGCAACAACTCTAAACG	439	CP
	RSDaV-R	GGACTGTCTGGAGGTAGCTCTTA		
Rose cryptic virus 1 (RoCV-1)	RoCV1-R1-252-F	GCGAGGAGTGCGTAATACATATAG	557	dsRNA1: RdRp
	RoCV1-R1-808-R	GCGCATGAATTGCTGGATAAG		
	RoCV1-R3-F	CGACTCCAATGCTAACGGATGA	762	dsRNA3: CP
	RoCV1-R3-R	CAGCAGCAGTAGCCAGAAATAG		
Rose partitivirus	RoParti-R1-956-F	GACTGGAGCAGATTCGATAAGG	614	dsRNA1:

(RoPV)	RoParti-R1-1569-R	CGAGCCATAAGTGATGGTAGAG		RdRp
Rose virus B (RVB)	RVB-F	GGTGTGCCTGAGAAGTCTATTG	361	CP
	RVB-R	GCTCTTCCTTTGTGGGTGATCTG		
Rose leaf rosette-associated virus (RLRaV)	RLRaV-F1	GCTGAGGGAAGCGTTGTTCCT	444	p58
	RLRaV-R1	CGTCTACCCTTCTTACCAACTC		
	RLRaV-F2	GCTTTACAGGATGGCTCTACTC	746	CP-p19
	RLRaV-R2	CAGCGTTACTCCACTCCATAAGC		
	RLRaV-F3	GTGGTGGATAACGACACTACTC	678	p58
	RLRaV-R3	GCTCTCACACGCATCTCACTATC		
	RLRaV-F4	TTTCCACATTGTAGGCTGTACTCC	300	ORF1a
	RLRaV-R4	CAACTCCCTAACCGCCTTATCT		
Plasmopara viticola lesion associated ourmia-like virus 33 (PvLaOV-33)	Ourmia-F	CTGCAGTAAGTGGGTCAATCTC	495	RdRp
	Ourmia-R	GATGCCCGTACGAAGGTAAGCA		

[a] Scott *et al.*, 1998.

[b] Malinowski and Komorowska, 1998.
