# Supplementary material for: Rose Virome Analysis and Identification of a Novel Ilarvirus in Taiwan
Source: Viruses. 2022 Nov 16;14(11):2537. doi: 10.3390/v14112537 (PMC9693529; doi:10.3390/v14112537)
Supplement: Supplementary file 1 [file viruses-14-02537-s001.zip › Table S2_Primers for RIV-2 genome sequencing-R1.pdf]

Table S2. Primers used for genome sequencing of rose ilarvirus 2.

Primer name	Sequence (5' → 3')	Position
RNA1		
RIV2-R1-27F	CGACCCGGATTAATCAGTTTTCT	27-49
RIV2-R1-3255R	ACATCCCAGAGGACCCCTTACT	3234-3255
RIV2-R1-2688F	CACATTGAAACACCCCTCTATCA	2688-2710
RIV2-R1-3052F	GCGGGATCTTCACACAACTTAG	3052-3073
RIV2-R1-577R	AACGCAACTCTCTCTCTGGTTGT	555-577
RIV2-R1-343R	CGTGTGAAGAAGTAGCTGAGTTG	321-343
RNA2		
RIV2-R2-234F	CGTTAGTGGAGAAGCTATCATGTG	234-257
RIV2-R2-2911R	CAAAGGTGGGCATATGGACTTAG	2889-2911
RIV2-R2-2412F	CGTTTCATGGACGTCACCTAGTA	2412-2434
RIV2-R2-2626F	GTCGTGGAGTATTTCCTCCTATGG	2626-2649
RIV2-R2-616R	CAAAACGGCTAGACCAGACAGC	595-616
RIV2-R2-393R	GTATGCCTCTTCAGGCACATCTC	371-393
RNA3		
RIV2-R3-1F	CGCTGAAGCTGTTATCCTACCTGA	339-362
RIV2-R3-1R	TGGAGAAAGGGACGCAAGATAGAG	1395-1418
RIV2-R3-2F	GGAACTGTCAACCGCTTCGAGGA	1257-1279
RIV2-R3-2R	AGCGCAAAAGCAATGACAGTACGG	2104-2127
RIV2-R3-1837F	GCTCCTAATAGAGTCCGTGTGAAG	1837-1860
RIV2-R3-2036F	CTCCTCCAGACTTTCTTGTCTGTG	2036-2059
RIV2-R3-590R	GAGCGGGTATTTTCATCACACA	569-590
RIV2-R3-441R	GGTCAAGACGACAGGCTTAGTT	420-441
