# Supplementary material for: Rose Virome Analysis and Identification of a Novel Ilarvirus in Taiwan
Source: Viruses. 2022 Nov 16;14(11):2537. doi: 10.3390/v14112537 (PMC9693529; doi:10.3390/v14112537)
Supplement: Supplementary file 1 [file viruses-14-02537-s001.zip › Table S3_Acc no_Ilarvirus.pdf]

Table S3. GenBank accession codes for genome sequences of *Ilarvirus* species analyzed in this study.

**Virus name**	**Abbrev.**	**RNA1**	**RNA2**	**RNA3**
Ageratum latent virus	AgLV	JX463340	JX463341	JX463342
American plum line pattern virus	APLPV	AF235033	AF235165	AF235166
Apple mosaic virus	ApMV	AF174584	AF174585	U15608
Asparagus virus 2	AV-2	EU919666	EU919667	X86352
Blackberry chlorotic ringspot virus	BCRV	DQ091193	DQ091194	DQ091195
Blueberry shock virus	BlShV	KF031037	KF031038	KF031039
Citrus leaf rugose virus	CLRV	U23715	U17726	U17390
Citrus variegation virus	CVV	EF584664	EF584665	U17389
Elm mottle virus	EMoV	U57047	U34050	U85399
Fragaria chiloensis latent virus	FClLV	AY682102	AY707771	AY707772
Humulus japonicus latent virus	HJLV	AY500236	AY500237	AY500238
Lilac ring mottle virus	LiRMoV	-[a]	EU919669	U17391
Lilac leaf chlorosis virus	LLCV	HE572565	FN669168	FN669169
Prune dwarf virus	PDV	U57648	AF277662	L28145
Parietaria mottle virus	PMoV	AY496068	AY496069	U35145
Prunus necrotic ringspot virus	PNRSV	AF278534	AF278535	U57046
Privet ringspot virus	PrRSV	KT290039	KT290040	KT290041

Prunus virus I	PrVI	MW579753	MW579754	MW579755
Rosa ilarvirus-1	RIV-1	MT017861	MT017862	MT017863
Rose ilarvirus 2	RIV-2	ON843765	ON843766	ON843767
Strawberry necrotic shock virus	SNSV	DQ318818	AY743591	AY363228
Spinach latent virus	SpLV	U93192	U93193	U93194
Tulare apple mosaic virus	TAMV	AF226160	AF226161	AF226162
Tomato necrotic streak virus	TomNSV	KT779204	KT779205	KT779206
Tobacco streak virus	TSV	U80934	U75538	X00435
Peanut stunt virus[b]	PSV	U15728	U15729	U15730

[a] The sequence is unavailable in the GenBank database.

[b] The virus belonging to the genus *Cucumovirus* of the family *Bromoviridae* was used as an out group.
