# Supplementary material for: Rose Virome Analysis and Identification of a Novel Ilarvirus in Taiwan
Source: Viruses. 2022 Nov 16;14(11):2537. doi: 10.3390/v14112537 (PMC9693529; doi:10.3390/v14112537)
Supplement: Supplementary file 1 [file viruses-14-02537-s001.zip › Table S4_Virus infection.pdf]

Table S4. Viruses were detected in the rose samples shown in Fig. 1.

Sample	ApMV	PNRSV	RIV-2	RoPV	ASGV	RSDaV
A	✓		✓		✓	
B	✓	✓	✓	✓		
C	✓	✓	✓	✓		
D	✓	✓	✓		✓	✓
E		✓				
F			✓			✓
G			✓			
H			✓			
